# Conducting correlation seminars in basic sciences at KIST Medical College, Nepal

**DOI:** 10.3352/jeehp.2011.8.10

**Published:** 2011-10-17

**Authors:** P. Ravi Shankar

**Affiliations:** Department of Medical Education, KIST Medical College, Lalitpur, Nepal.

**Keywords:** Educational measurement, Learning, Medical school, Nepal, Undergraduate medical education

## Abstract

KIST Medical College is a new medical school in Lalitpur, Nepal. In Nepal, six basic science subjects are taught together in an integrated organ system-based manner with early clinical exposure and community medicine. Correlation seminars are conducted at the end of covering each organ system. The topics are decided by the core academic group (consisting of members from each basic science department, the Department of Community Medicine, the academic director, and the clinical and program coordinators) considering the public health importance of the condition and its ability to include learning objectives from a maximum number of subjects. The learning objectives are decided by individual departments and finalized after the meeting of the core group. There are two student coordinators for each seminar and an evaluation group evaluates each seminar and presenter. Correlation seminars help students revise the organ system covered and understand its clinical importance, promote teamwork and organization, and supports active learning. Correlation seminars should be considered as a learning modality by other medical schools.

In Nepal, a developing country in South Asia, the undergraduate medical (Bachelor of Medicine and Bachelor of Surgery, MBBS) course is of four and half years duration followed by one year of compulsory rotating internship. The basic science subjects of anatomy, physiology, biochemistry, pathology, microbiology, and pharmacology are taught in an integrated organ system-based manner during the first two years of the course. KIST Medical College (KISTMC) is a new medical school in the Lalitpur district of the Kathmandu Valley committed to excellence in holistic healthcare, education, and research. The first class of students was admitted to its program in November 2008 and the third class was admitted in November 2010.

The college is affiliated with the Institute of Medicine (IOM) at Tribhuvan University for the MBBS course. The curriculum stresses community-based learning and early clinical exposure [1]. The topics covered in the first year are basic concepts and the musculoskeletal and neurosensory organ systems. The organ systems covered during the second year are the respiratory, cardiovascular, gastrointestinal, renal and electrolyte, endocrine, and reproductive systems. Students visit the hospital for early clinical exposure for four hours once a week. The emphasis is on history-taking during the first year and physical examination during the second year. The institution stresses self-directed, active learning by students. The predominant teaching-learning methodology is didactic lectures, but problem-based small group learning sessions are conducted in pharmacology [2]. A medical humanities module named 'Sparshanam' is also conducted for first year students [3].

Correlation seminars in basic sciences are an approach that brings together learning objectives from different basic science subjects, community medicine, and relevant clinical subjects using a common/prevalent disease condition within a particular organ system. In Nepal, correlation seminars were initiated at the IOM, the oldest medical college in the country, to promote integrated, active learning among students and highlight the importance of basic sciences in clinical medicine. At Duke University in the United States, a program to integrate cognitive and affective approaches into medical education was tried [4]. Clinical correlation seminars, lectures, discussions, clinical clerkships and medical humanities were components of the integration program. Seminars are also regularly conducted at Manipal College of Medical Sciences, Pokhara, Nepal [5], but they may not always be integrated with the subject matter or organ system being covered during the theory classes. Seminars are held once every fortnight. At the BP Koirala Institute of Health Sciences, Dharan, student seminars are conducted as a part of the week long problem-based learning package [6]. To the author's personal knowledge, seminars have also been conducted in other medical schools, but descriptions of these seminars have not been available in the literature. In a previous article, the author described correlation seminars as a method of integrated learning at KISTMC [7]. In this article, he describes correlation seminars at KISTMC in greater detail.

Correlation seminars are conducted at the end of covering each organ system during the first and second year. During a correlation seminar, a common disease or problem involving the organ system just covered is selected. The college has created an academic core group consisting of faculty members from each of the basic science departments, community medicine, the academic director, and the clinical and program coordinators. The topics for correlation seminars are selected following discussion and deliberation among group members. Topics are selected based on the importance of the condition, its public health relevance, and its ability to integrate learning objectives from the maximum number of subjects. Following the selection of topics, departments are asked to generate two or three learning objectives for each seminar topic, if possible. Some departments may not be able to generate a learning objective in relation to certain topics. After three or four days, there is another meeting of the academic core group where the objectives are discussed and modified as required. The wording of the objectives receives careful attention to ensure they are as objective and unambiguous as possible. Previously, one topic was selected for each organ system, but from November 2010 onward, three topics have been chosen to involve a greater number of students in the seminars. For the most recent of the correlation seminars on the gastrointestinal system, the conditions selected were peptic ulcer, diarrhea, and hepatitis. Table 1 shows the different topics selected for the various organ systems in the first and second years in which correlation seminar were conducted. Table 2 shows the learning objectives developed for the topic peptic ulcer, a very common disease in Nepal.

After learning objectives have been framed and agreed upon by faculty members, they are distributed to students by the program coordinator. The college admits 100 students to the MBBS course each year. As between 40 to 50 learning objectives are framed for each set of correlation seminars, each objective is distributed to a small group consisting of two to three students. We also select two students as overall coordinators for the seminar. These students have the responsibility of organizing the seminar in an appropriate sequence, introducing different topics and ensuring proper execution of the seminar. All students who are assigned a particular learning objective prepare for a five minute presentation of that objective using the college library, text books, and help from faculty members of the relevant departments. Students use assigned textbooks, reference books available in the library, notes from faculty lectures, and articles and images obtained through Google searches. We stress the importance of citing the source of their material on their slides to make them aware of plagiarism and intellectual property rights. The students have found the college library with textbooks, reference books, and access to biomedical journals through HINARI to be adequate. Students assigned clinical objectives obtain help from relevant clinical departments. The final presentations are approved by faculty members of different departments.

An evaluation group consisting of one faculty member from a basic science department and one faculty member from the department of community medicine is formed for each of the correlation seminars. A senior faculty member is entrusted the responsibility of being the team leader. Each member of the evaluation team assesses each student presenter. The student presenters are selected on the basis of their past record of presentations by using a draw of lots. Students who have not presented before or have presented a smaller number of times are usually selected. The assessment of the student presenters includes the appearance of the presenter and the slides, clear presentation of objectives, interest and enthusiasm, audiovisual aids, voice, organization of the presentation, adhering to the allotted time, summarizing the presentation, answering questions, and an overall grade for the presentation. For each of these categories, the evaluator is required to choose one of five values: 0, 0.25, 0.5, 0.75 or 1 (with 0 representing the worst performance and 1 the best). The mean grading of different evaluators is calculated and is made available to the student presenter for feedback and further improvement. The overall scores have been satisfactory up to the present time. The scores are added to the formative assessment of students. The team leader also provides a brief 100-word report on the seminar, which is made available to all students.

Most students present using Microsoft PowerPoint slides. Despite encouragement to use them, use of an overhead projector and white board is not common. Students have often been interested in using slides similar to those used by faculty members during their lectures. Faculty members from each department are required to check and comment on the slides prepared by students and approve them before they are used in the final presentation. In about 20% of cases this checking procedure has not been strictly followed by both students and faculty. Many objectives used during seminars were not covered in detail during theory classes. A certain degree of academic plagiarism continues despite our efforts, and could be considered a weakness of the correlation seminars. One of the problems noted has been the use of slides with diverse backgrounds, some of which were noisy and not easily readable. To ensure the uniformity of presentations, we have recently developed guidelines for presentation. Informal feedback obtained from students suggests that correlation seminars have been effective in promoting self-learning and active learning among students. Students also seem to obtain a holistic view of the disease and understand its clinical implications. We plan to collect formal feedback in the future.

Correlation seminars help students review the organ system covered in lectures and understand its clinical importance, promote team work and organization, and support active learning. Correlation seminars should be considered as a learning modality by other medical schools in south Asia.

## Figures and Tables

**Table 1:**
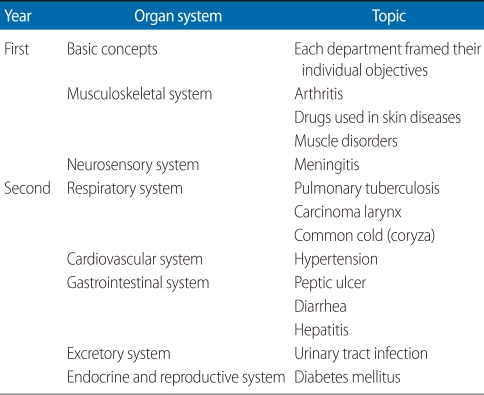
Correlation seminar topics selected for different organ systems

**Table 2:**
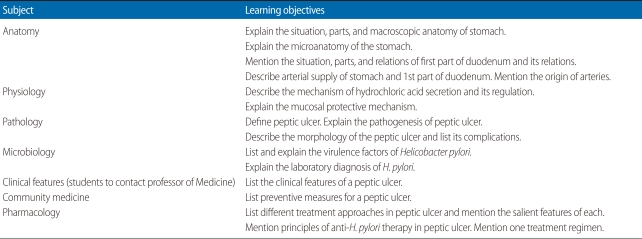
Learning objectives for the topic "peptic ulcer"
